# Hepatitis C and hepatitis B virus infection in hemodialysis patients after nationwide direct antiviral agents therapy—experience of 10 Romanian HD centers

**DOI:** 10.1007/s11255-023-03587-0

**Published:** 2023-04-06

**Authors:** Luciana Marc, Adelina Mihaescu, Raluca Lupusoru, Oana Schiller, Flaviu Bob, Lazar Chisavu, Felix Bende, Roxana Sirli, Adalbert Schiller

**Affiliations:** 1 Department of Internal Medicine II – Division of Nephrology, “Victor Babeș” University of Medicine and Pharmacy, 300041 Timisoara, Romania; 2 Center for Molecular Research in Nephrology and Vascular Disease, Faculty of Medicine, “Victor Babeș” University of Medicine and Pharmacy, 300041 Timișoara, Romania; 3 Advanced Regional Research Center in Gastroenterology and Hepatology, Department of Internal Medicine II – Division of Gastroenterology and Hepatology, “Victor Babes” University of Medicine and Pharmacy, 300041 Timisoara, Romania; 4 Center for Modeling Biological Systems and Data Analysis, Department of Functional Sciences, “Victor Babes” University of Medicine and Pharmacy, 300041 Timisoara, Romania; 5 Nephrology Clinic - County Emergency Hospital “Pius Brinzeu”, Timisoara, Romania; 6 Gastroenterology and Hepatology Clinic - County Emergency Hospital “Pius Brinzeu”, Timisoara, Romania; 7 “Avitum” Center of Hemodialysis, Timisoara, Romania

**Keywords:** ESKD, Hemodialysis, Hepatitis C virus, Hepatitis B virus, DAA

## Abstract

**Purpose:**

End-stage kidney disease patients (ESKD) receiving hemodialysis (HD) are at a greater risk of hepatitis virus (HV) infections due to the invasive nature of the procedures, frequent hospital stays and surgeries, as well as the immune deficiency status of ESKD.

**The aim:**

This study was to reassess the hepatitis virus infections prevalence in the HD population in Romania after 5 years of oral DAAs therapy and assess the impact on HD patients’ outcomes in two cohorts (2015 and 2019).

**Methods:**

We compared ESKD patients treated with HD in 10 HD centers from the historical regions of Romania in 2015 (*n* = 1401, Mean age 59.7 ± 12.92 years) with patients treated in the same centers in 2019 (*n* = 1698, mean age 61 ± 12.93 years). All patients went through HD therapy for more than 90 days.

**Results:**

The patients from the 2019 cohort were significantly older (*p* = 0.005), had a longer duration of HD therapy (*p* < 0.0001), and had more vascular calcifications (*p* = 0.015); the crude one-year mortality rate did not differ from the 2015 cohort (9.9 vs. 10.7%, *p* = 0.46). The prevalence of HBV infection did not differ between the cohorts (4.7% vs. 4.8, *p* = 0.604) but the prevalence of HCV significantly decreased from 2015 to 2019 (16.9 vs. 10.5%, *p* < 0.0001).

**Conclusion:**

After 15 years of a nationwide infection prevention program for HV infections and 5 years of DAAs treatment in Romania, the prevalence of HBV did not change but HCV infections decreased significantly, however, it still remained high.

**Supplementary Information:**

The online version contains supplementary material available at 10.1007/s11255-023-03587-0.

## Introduction

The high risk of hepatitis virus (HV) infections in patients with end-stage kidney disease (ESKD) treated with hemodialysis (HD) is dependent on the HV infection epidemiology in the general population of the area, the need for frequent hospital stays and surgeries of these patients as well as the immune deficiency status of ESKD. HV infections are endemic in South-East Europe. According to ECDC (European Centre for Disease Prevention and Control) data, from 2016, the prevalence of HBV infection in Romania was estimated to be 4.4–6.2% and of HCV 3.2% [1]. Until 1999, in Romania, blood donors have not been tested for HV infection and most of the ESKD patients were treated for renal anemia with iron therapy and blood transfusion. In 2000 a complex prevention program was implemented nationwide in Romania to reduce HV infections epidemiology in HD centers and in the general population. After 15 years of systematically applied prevention programs, the prevalence of HV infections in HD centers decreased from 17 to 21.6% (2000) to 9.5% for HBV infections (2015) and from 28.6 to 75% (2000) to 27.3% for HCV infection (2015). In 2015, 5% of the patients presented with both HBV and HCV infection [2–4]. In 2015 the oral, direct-acting antivirals (DAAs) were implemented in Romania for the treatment of HCV infections, supported by a program of the health insurance system and the Ministry of Health. The novel DAAs had little or no restriction for treating patients with severe CKD or ESKD so patients treated with HD could also be included in these programs.

*The aim* of this study was to reassess the hepatitis virus infections prevalence in the HD population in Romania after 5 years of oral DAAs therapy as well as to assess the impact of DAAs on the outcome of HD patients.

## Material and methods

We compared ESKD patients treated with HD in 10 HD centers from the historical regions of Romania (Moldova—Eastern, Muntenia–Southern, Transylvania—Centre and Banat—Western Romania) in 2015 (*n* = 1401, Mean age 59.7 ± 12.92 years, 818 men, 583 women) with patients treated in the same centers in 2019 (*n* = 1698, mean age 61 ± 12.93 years, 1014 men and 684 women). We also traced and assessed the survivals from 2015 to 2019.

All patients received HD therapy for more than 90 days. The number of patients is significant in both cohorts for the ESKD patients treated with HD in Romania. Personal data, medical history, duration of dialysis, comorbidities and lab results (HBs Ag and anti-HCV antibodies, hemoglobin, ferritin, transferrin saturation, calcium, phosphorus, intact parathyroid hormone (iPTH), vitamin D and albumin) have been retrieved from the patient’s personal files and their medical records. The assessed comorbid conditions were: diabetes mellitus (DM), coronary artery disease, left ventricular hypertrophy, heart valve calcifications, peripheric artery disease, and a history of stroke. The study was retrospective observational and each cohort was followed up for twelve months to assess one-year mortality. Patients with renal transplants, patients transferred to other HD facilities during the twelve months follow-up, or the ones who switched to other renal replacement therapies as well as the patients treated for less than 3 months were excluded from each of the two cohorts. HD therapy was performed using high-flux, high-surface, single-use polysulfone filters and B. Braun acidic bicarbonate hemodialysis concentrate. HD was performed on the 2015 cohort by catheters in 16.48% of cases, an arteriovenous graft in 1.95% and an arteriovenous fistula in the remaining cases. In the 2019 cohort, dialysis was performed using catheters in 21.99% of cases, arteriovenous graft in 0.52% and arteriovenous fistula in the remaining cases. „No touch” catheter handling protocols have been applied and surface sterilization after each HD session was routinely used. HV infected patients have been treated in isolated rooms with separate dedicated machines (for HBV, HCV and HB + CV infected patients). Staff members were negative for HV infection markers. The protocol presented above was implemented nationwide in 2004 for the prevention of HV infections and applied since [5].

All HV-infected patients accepting DAAs therapy have been treated according to the first Romanian recommendation guideline developed by the Romanian Society of Gastroenterology and Hepatology (SRGH) [6].

All patients gave their written informed consent for the handling of medical data for medical research purposes and the study was conducted according to the Helsinki declaration, bearing the approval of the ethics committee of the dialysis centers and of the “Victor Babes” University of Medicine and Pharmacy, Timisoara, Romania nr.43/2017.

### Statistical analysis

Data analysis was performed using MedCalc software v19.3. Data is presented as mean ± standard.deviations for continuous variables with Gaussian distribution, median (interquartile range) forcontinuous variables without Gaussian distribution or percentages for categorical variables. The lowerand upper limits of the 95% confidence intervals (CI), used to estimate the prevalence, were calculatedaccording to Wilson’s procedure for variables with Poisson distribution. Categorical variablecomparisons were performed using the chi-square or Fisher exact test, and continuous variables wereevaluated with Student’s t test or Mann–Whitney test. A p-value < 0.05 as used for significance. The Kaplan–Meier analysis was used to ass ss all-cause mortality according to HV infection status and a Cox proportional hazards regression mod l was built to assess what risk factors influence 1-year survival.

## Results

The general data of the two cohorts are highlighted in Table 1.

**Table 1:** Comparison between 2015 and 2019 cohorts

Parameter	2015 (*n* = 1401)	2019 (*n* = 1698)	*p*-value
Age (years)	59.7 ± 12.92	61 ± 12.93	0.0054
Gender (male)	818 (58.3%)	1014 (59.71%)	0.43
Duration of HD therapy (years)	4.73 ± 4.16	5.57 ± 4.66	< 0.0001
Type 2 diabetes mellitus	369 (26.33%)	432 (25.44%)	0.56
Coronary artery disease	997 (71.16%)	843 (55.57%)	< 0.0001
Left ventricular hypertrophy	947 (67.59%)	1139 (75.08%)	< 0.0001
Peripheric artery disease	381 (27.19%)	376 (24.78%)	0.128
Stroke history	284 (20.27%)	160 (10.5%)	< 0.0001
Hemoglobin (g/dl)	10.95 ± 1.46	10.3 ± 1.45	< 0.0001
Ferritin (ng/ml)	1042.4 ± 782.8	917.36 ± 625.0	< 0.0001
TSAT (in %)	36 ± 31.7	33.65 ± 48.86	0.113
CRP (mg/dl)	12.2 ± 67.9	15.5 ± 30.4	0.071
Phosphorus (mg/dl)	5.1 ± 1.55	5.04 ± 1.43	0.06
Calcium (mg/dl)	8.9 ± 1.43	8.77 ± 0.72	0.0011
iPTH (pg/ml)	456 ± 540.5	546.42 ± 576.5	< 0.0001
Albumin (g/dl)	3.97 ± 1.34	4.06 ± 0.43	0.0091
HBV (HBs antigen)	66 (4.7%)	83 (4.8.%)	0.604
HCV (Anti HCV antibody)	237 (16.9%)	179 (10.5%)	< 0.0001
Parathyroidectomy (%)	86 (6.81%)	154 (9.06%)	0.024
Heart valve calcification (%)	961 (68.59%)	1100 (72.51%)	0.015
Crude 1 year mortality rate (%)	9.9%	10.7%	0.466

*CRP* reactive C protein, *HD* hemodialysis, *TSAT* Transferrin saturation, *HBV* hepatitis B virus, *HCV* hepatitis C virus, *SD* standard deviation

Though the patients from 2019 were significantly older (*p* = 0.005), had a longer duration of HD therapy (*p* < 0.0001), had a higher prevalence of LVH (*p* < 0.0001), higher average iPTH and lower Ca levels (*p* = 0.001) and more vascular calcifications (*p* = 0.015), the crude one-year mortality rate did not differ from the 2015 cohort (9.9% vs. 10.7%, *p* = 0.46) (Table 1). These results may be related to the fact that the type 2 diabetes mellitus (T2DM) prevalence did not significantly differ between the cohorts, and the patients from 2019 had a lower prevalence of coronary artery disease, history of stroke and higher average hemoglobin and albumin levels (see Table 1).

The prevalence of HBV infection did not differ between the cohorts (4.7% vs. 4.8, *p* = 0.604) but the prevalence of HCV significantly decreased from 2015 to 2019 (16.9 vs. 10.5%, *p* < 0.0001). The HV infection prevalence differed between the different investigated geographic areas. HBV-infected patients were more common in Eastern and Central Romania in both cohorts. Both cohorts reported HCV antibody-positive patients were more prevalent in Moldova and Muntenia, while prevalence significantly decreased from 2015 to 2019 (Table 2).

**Table 2:** HV infection prevalence in both cohorts and in the investigated areas

	Overall		Banat		Moldova		Muntenia		Transilvania	
			(2015, *n* = 435)		(2015, *n* = 405)		(2015, *n* = 164)		(2015, *n* = 397)	
			(2019, *n* = 517)		(2019, *n* = 495)		(2019, *n* = 192)		(2019, *n* = 493)	
	HBV	HCV	HBV	HCV	HBV	HCV	HBV	HCV	HBV	HCV
2015	66	237	16	51	23	113	4	29	23	44
(*N* = 1401)	(4.7%)	(16.9%)	(3.6%)	(11.7%)	(5.6%)	(27.9%)	(2.4%)	(17.6%)	(5.7%)	(11%)
2019	83	179	24	38	25	92	7	15	27	34
(*N* = 1697)	(4.8%)	(10.5%)	(4.6%)	(7.3%)	(5%)	(18.5%)	(3.6%)	(7.8%)	(5.4%)	(6.8%)
*p*-value	0.896	< 0.0001	0.650	0.020	0.688	0.0008	0.511	0.005	0.845	0.027

We also compared in both cohorts, the patients with and without HV infection. In 2015 the patients with HV infection had a significantly longer average dialysis therapy duration (7.7 vs. 3.9 years, *p* < 0.0001), lower prevalence of Type 2 DM (21 vs. 27.7%, *p* = 0.021), lower prevalence of coronary artery disease (43.1 vs. 69.4%, *p* < 0.0001), though the higher prevalence of heart valve calcification (77.8 vs 66.2% *p* < 0.001), higher average Hb levels (11.2 vs 10.8 g/dl, *p* < 0.0001), higher iPTH average values (535.5 vs. 436.2 pg/ml, *p* = 0.005) and more parathyroidectomy (9.7 vs 5.1%, *p* = 0.003), however, average calcium and phosphate levels did not differ. Average albumin level and one-year crude death rate did not differ either (12.2 vs. 9.4%, *p* = 0.158).

In the 2019 cohort HV positive patients presented longer average dialysis therapy duration (8.5 vs. 5 years, *p* < 0.0001), even lower prevalence of Type 2 DM (17.6 vs. 27.6%, *p* = 0.002), higher prevalence of coronary artery disease (71.6 vs. 48.8%, *p* < 0.0001) and left ventricular hypertrophy (72.4 vs. 66.1 *p* = 0.050). More parathyroidectomy was performed (15.3 vs. 8%, *p* = 0.0003), prevalence of heart valve calcification was higher (70.4 vs. 63.8%, *p* = 0.044) and the average iPTH value was also elevated (621 vs. 533.4 pg/ml, *p* = 0.025) even if calcium and phosphate average values did not differ in these patients. In HV-positive patients less inflammation was registered i.e., lower average CRP (1.1 vs. 1.6, *p* < 0.05) and Ferritin (839 vs.930 ng/ml, *p* < 0.05) levels were detected. Lower average albumin level (4 vs. 4.1 g/dl, *p* < 0.001) and lower one-year crude death rate (6.8 vs. 11.3%, *p* < 0.05) were registered in these patients. (Supplementary tables 1 and 2).

**Fig. 1:**
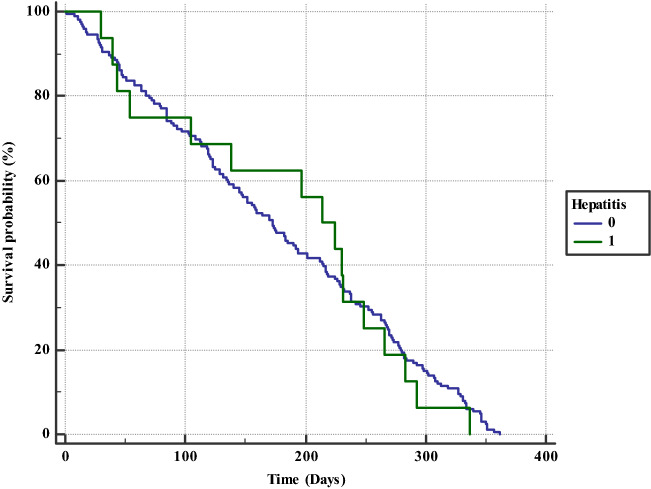
Kaplan–Meier analysis for all-cause mortality according to hepatitis virus infection status (infected versus non-infected) in 2019 cohort

**Fig. 2:**
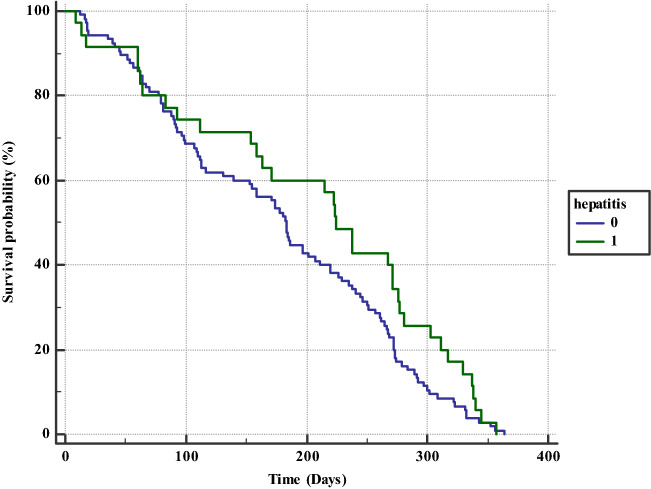
Kaplan–Meier analysis for all-cause mortality according to hepatitis virus infection status (infected versus non-infected) in 2015 cohort

One-year survival analysis.The overall mortalities rates in the 2019 and 2015 cohorts were similar, but there were differences between the mortality of HV positives and negatives in each cohort. In the 2015 cohort, the mortality difference was statistically insignificant (9.7 vs 12.3%, *p* = 0.19). In the 2019 cohort, the mortality among the HV infected was significantly lower, compared to thosewho were not infected (6.8% vs 12.9, *p* = 0.006). Figures 1 and 2 illustrate the Kaplan-Maier survival curves for the 2015 and 2019 cohorts, respectively.

Age, HCV infection and comorbidities were associated with a poor prognosis, as demonstrated in the multivariate cox regression model (Tables 3 and 4).

**Table 3:** Cox Regression model for the 2015 cohort

Parameter	Hazard ratio	95% CI	Standard error	*p*-value
Age	1.03	1.01–1.05	0.0007	< 0.0001
Albumin	1.03	0.97–1.10	0.03	0.20
HCV	1.46	0.97–2.18	0.20	0.04
HBV	1.01	0.44–2.29	0.41	0.97
Vitamin D	0.94	0.94–0.97	0.12	< 0.0001
Comorbidities	1.43	1.01–2.04	0.17	0.04

**Table 4:** Cox Regression model for the 2019 cohort

Parameter	Hazard ratio	95% CI	Standard error	*p*-value
Age	1.02	1.00–1.03	0.006	0.0007
Albumin	0.33	0.28–0.39	0.07	< 0.0001
HCV	0.44	0.23–0.83	0.32	0.01
HBV	1.01	0.47–2.16	0.38	0.97
Vitamin D	0.98	0.89–1.03	0.005	0.58
Comorbidities	1.01	0.99–1.98	0.35	0.07

The mortality risk in the 2015 cohort was significantly influenced by: age (HR 1.03, *p* < 0.0001), comorbidities (HR 1.43, *p* = 0.04), and HCV (HR = 1.46, *p* = 0.04). According to the Cox regression model, Tables 3 and 4 show that the death risk was not increased by serum albumin and vitamin D status.

The mortality risk in the 2019 cohort was significantly influenced only by age (HR 1.02, *p* = 0.0007).

Of the 66 HBV-infected patients (2015) in the five years, 42.4% (28cases) initiated antiviral therapy according to the Romanian guidelines and indication of the gastroenterologist (Tenofovir alafenamide or Tenofovir disoproxil fumarate as available). The compliance to therapy was poor due mainly to personal reasons (therapy too long, deaths among known persons, attributed to therapy side effects).

None of the patients was treated for more than 18 months despite medical recommendations and the evolution of the viral load was unavailable. In the traced five years of survival two new cases have been registered, and 45 new entry patients in the 2019 cohort presented HBV infection. So, the prevalence of HBV-infected patients did not significantly increase but the absolute number, yes.

Of 237 HCV-infected patients in 2015, 20.2% (48) accepted DAA therapy according to the Romanian guidelines (see Discussion), and all patients had an SVR (sustained virologic response) at the end of the control period. One patient presented relapse/reinfection (severe liver cirrhosis) and died shortly after.

Six hundred nineteen patients from the 2015 cohort could be traced to the 2019 cohort representing 44.1% survival after 5 years of HD therapy. 36 out of the 66 HBV-infected patients in 2015 survived (5 years mortality was 45.4%. Out of the 48 HCV patients treated with DAA until 2019 20.8% (10) died. Of the 191 (79.8%) HCV antibody-positive patients, not treated, in the five years period 121 (63.3%) died. Of the not infected patients in 2015, in the 5 years, 55.7% died.

In the five years (2015–2019), in the 10 centers, 1079 new entries have been registered (an average of 21.5 patients per center/year). Of these patients, 73 were positive for HCV antibodies representing 6.7% of the new-entry patients.

## Discussion

End-stage kidney disease patients (ESKD) represent an important group at risk for HBV and HCV infections. Besides the common risk for transmission (needle for other than medical purposes and sexual transmission), patients with ESKD are exposed to a high risk of HV infection by nosocomial and iatrogenic routes (frequent hospital admission, blood transfusion or other human origin substances, diabetes, dialysis). In our country, strategies for infection control against blood-borne pathogens are implemented in an effort to reduce the rate of HV infection. All of these protocols are extensively described in the methods section. The ECDC estimated in 2016 that patients treated with HD in Romania have an HBV infection prevalence in HD patients of 7.91–9.5% and an HCV prevalence of 27.3–39.3% [1]. According to our data (the 2015 cohort–1401 HD patients in 10 HD centers from all geographic areas of Romania) the mean prevalence of HBV infection was 4.7% (between 2.4 and 5.6%) and of HCV infection was 16.9% (between 11 and 27.9%). The differences between ECDC and our results may be explained by the fact that ECDC used for estimations of older data (from 2010) and a smaller number of cases. The mean prevalence of HBV infection remained the same in the 2019 cohort, 4.8% (between 3.6 and 5.4%), but the absolute number increased due mainly to new entries before the 2019 cross-section analysis. As we have evidenced before, for personal reasons and despite all medical advice, the adherence to anti-HBV therapy was inferior (al patients abandoned therapy before completion).

After 5 years of the DAA therapy nationwide program, the prevalence of positive anti-HCV antibody patients decreased to 10.5% (between 6.8 and 18.5%). In HCV infection, HCV antibodies can be detected even if the viral load becomes negative under DAA therapy. Therefore, the decreased prevalence of HCV antibody-positive patients should be attributed to other causes (death, HCV-negative new entries, less active infections in the general population and exclusion from the group of HCV antibody-positive patients of those tested RNA-negative after DAA therapy). The number of patients accepting DAA therapy was low (20.2% of infected patients in 2015), though all patients after the mandatory follow-up period (and 3 PCR determinations) became negative. One should not forget that the DAA program was addressed to the infected general population and to other high-risk groups, so one should expect a decrease in infected new entries in HD. Data about the decrease in HCV infection prevalence and incidence in the HD centers of DOPPS (Dialysis Outcome and Practice Patterns Study) was recently published. It seems that prevalence decreased from 14 to 8% and the incidence from 2.9 to 1.2 cases/100 patient years but nosocomial transmission in the HD units still remained important [7].

The influence of HBV and HCV infection on mortality in the general population is well documented. In 2015, 96% of 1.3 million deaths caused by hepatitis viruses worldwide were attributed to HBV and HCV and more than 50% were related to viral-induced cirrhosis [8]. Less information is available concerning HV infection influence on the mortality of ESKD patients treated with HD. Most of the available data supports the increased risk of mortality in HD-treated patients with HCV infection before DAA therapies. In the United States (2007) HCV infection was associated with increased risk of all-cause and cardiovascular mortality in almost all clinical, demographic and laboratory groups of patients (HR 1.25 (95% CI 1.12–1.39) [9]. In 2009 in Japan HCV infection was highly predictive for mortality (RR 1.37, 95% CI 1.15–1.62, *p* = 0.003), similar results registered in Australia and New Zealand (adjusted HR 1.29, 95% CI 1.05–1.58) [10]. An interesting result was published based on the Sicilian Registry of Hemodialysis and Transplantation (2009). HCV infection increased mortality risk in HD patients but only in women aged < 65 years (OR 1.77 95% CI 1.12–2.79) and the results suggested a link to HCV increase of cardiovascular morbidity and mortality in that group of patients [11]. In our 2015 cohort (before the introduction of DAA therapy in Romania) the risk of mortality was increased by the presence of HCV antibodies (HCV infection). In fact, the one-year mortality risk was influenced by age (HR 1.03, *p* < 0.0001), comorbidities (HR 1.43, *p* = 0.04) and the HCV infection (HR 1.46, *p* = 0.04).

The results regarding the influence of HBV infection on HD patients is less clear. The analysis of DOPPS data found a prevalence of HBV infection in HD centers ranging from 0% (United Kingdom) to 4.6% (Germany) in 2003 [12]. In a recent metanalysis from Africa, the prevalence of HBV infection was ranging between 4.09 and 12.27% in HD centers [13]. Complex infection prevention measures, vaccination and therapy significantly reduced the disease's prevalence in the general population and in HD patients (the prevalence of HBV infection in HD patients remains significantly higher than in the general population). In a previous paper, we investigated the prevalence of HBV infection in Romanian HD centers, in 2010, after over 10 years of complex infection prevention measures and HBV vaccination and our results evidenced a reduction in prevalence from 17 to 9.5% [4]. In our 2015 cohort, the prevalence of HBV infection further decreased to 4.7% and remained unchanged in the next 5 years, 4.8%. (2019 cohort). No effect of VHB infection on mortality was detected in an HD center in 1977 Philadelphia [14] or the Asia–Pacific area in 2009 (in 201 590 HD patients) [10]. In Korea, 2015, HVC but not HVB, increased the risk of mortality in HD [15]. In our 2015 cohort also HCV but not HBV infection increased mortality risk. By tracing the survivals of the 2015 cohort into the 2019 one, we evidenced a 44.1% (619 patients) 5 years survival rate. Actually, the survivors represent 36.4% of the 2019 cohort. We also determined a differential 5 years mortality for the HV-infected and not-infected patients. For the HBV-infected patients, the 5 years mortality turned out to be 45.4%. For the HCV patients with DAA therapy, it was 20.8%, for the HCV non-treated group it was 63.3% and for the non-infected patients it was 55.7%.

Until 2018 more than 18,000 patients have been treated with DAA (including ESKD patients from HD centers) with a sustained virologic response (SVR) obtained in around 99% of the cases [16].

In our 2019 cohort (after DAAs in patients who accepted treatment) the prevalence of HCV infection decreased in 5 years from 16.9 to 10.5% (*p* < 0.0001) but, as stated before, the prevalence of HBV infection did not change. Regarding mortality risk, data from recent reviews are controversial. Some studies indicate that the presence of HCV infection influences cardiovascular disease-related mortality in HD patients [21], while others imply that HCV infection in HD patients no longer increases cardiovascular mortality [22]. In our 2019 cohort, mortality risk was significantly influenced only by age (HR 1.02, *p* = 0.0007), When comparing the pooled HV-infected patients from the two cohorts it seems that after the 5 years DAA therapy program it was less inflammation among the 2019 infected group (CRP *p* < 0.0001, Ferritin *p* = 0.0001), higher prevalence of coronary artery disease (*p* < 0.0001), but a lower prevalence of peripheric artery disease (*p* = 0.009), stroke (*p* < 0.0001) and heart valve calcification (*p* = 0.035). In the same time, there was a significant difference concerning one-year mortality between the HV-infected group before DAA therapy (2015 cohort) and after DAA therapy (cohort 2019) (12.2 vs 6.8% *p* = 0.017).

In large population-based cohorts treated with DAA and associated with SVR, HCV-related mortality was significantly reduced [18], though restoration of immune response is only partial [19] and circulating CD-8 + T cells remain still dysfunctional, especially in advanced chronic liver disease [20]. After 5 years from SVR, the risk of relapse/reinfection is small, mainly in high-risk patients (HIV associated). The beneficial effect of DAA on liver pathology is under current assessment, the slowing of CKD progression in HCV-infected patients also. Less data is available about HCV-positive ESKD patients on HD therapy and hard end-point studies are still expected.

## Conclusion

After 15 years of nationwide infection prevention program for HV infections and 5 years of DAAs treatment in Romania, the C virus infections prevalence significantly decreased in the HD centers. However, it still remained high. In 2019 HCV virus infection was no longer associated with mortality. HBV infection prevalence, however, did not decrease in the last 5 years and it became associated with mortality risk in HD patients. To further decrease the HV infections burden in the general population and in HD patients more sustained efforts should be made in infection prevention, HBV vaccination and HBV/HVC therapy.

### Supplementary Information

Below is the link to the electronic supplementary material.

Supplementary file1 (DOCX 38 KB)

## Data Availability

The raw data supporting the conclusions of this article will be made available by the authors, without undue reservation.

## Declarations

### Human and animal participants

No animal studies are presented in this manuscript. The studies involving human participants were conducted in accordance with the Declaration of Helsinki and reviewed and approved by Victor Babes” University of Medicine and Pharmacy, Timisoara, Romania nr.43/2017. No potentially identifiable human images or data is presented in this study.
