# Supplementary material for: Hepatitis C and hepatitis B virus infection in hemodialysis patients after nationwide direct antiviral agents therapy—experience of 10 Romanian HD centers
Source: Int Urol Nephrol. 2023 Apr 6;55(11):2951–8. doi: 10.1007/s11255-023-03587-0 (PMC10560143; doi:10.1007/s11255-023-03587-0)
Supplement: Supplementary file 1 — Supplementary file1 (DOCX 38 KB) [file 11255_2023_3587_MOESM1_ESM.docx]

Supplementary Material

Table 1

| Parameter | HV- Infected  (N=286) | Non-Infected (N=1116) | P - value |
| --- | --- | --- | --- |
| Age (years) | 59.54 ± 12.65 | 59.74 ± 12.99 | 0.814 |
| Gender (male) | 53.3% (152) | 59.6% (665) | 0.054 |
| Duration of hemodialysis (years) | 7.79 ± 5.72 | 3.95 ± 3.22 | <0.0001 |
| Type 2 diabetes mellitus (%) | 21.05% (60) | 27.71% (309) | 0.021 |
| Coronary artery disease (%) | 43.15% (123) | 69.41% (774) | <0.0001 |
| Left ventricular hypertrophy (%) | 65.26% (186) | 69.5% (775) | 0.162 |
| Peripheric artery disease (%) | 31.22% (89) | 26.09% (291) | 0.077 |
| Heart valve calcification (%) | 77.89% (222) | 66.27% (739) | 0.0002 |
| Parathyroidectomy | 9.79% (28) | 5.19% (58) | 0.003 |
| Presence of stroke (%) | 23.85% (68) | 19.37% (216) | 0.091 |
| Hemoglobin (g/dl) | 11.25 ± 1.7 | 10.88 ± 1.3 | <0.0001 |
| Ferritin (ng/ml) | 1107.47 ± 884.6 | 1026.35 ± 755.3 | 0.118 |
| TAST (%) | 37.85 ± 17.6 | 34.89 ± 34.3 | 0.151 |
| PCR | 9.87 ± 24.5 | 12.60 ± 74.9 | 0.533 |
| Phosphorus (mg/dl) | 5.14 ± 1.4 | 5.13 ± 1.5 | 0,918 |
| Calcium (mg/dl) | 8.82 ± 0.9 | 8.92 ± 1.5 | 0,281 |
| iPTH (pg/ml) | 535.57 ± 616.7 | 436.2 ± 517.7 | 0.005 |
| Albumin (g/dl) | 3.84 ± 0.4 | 4 ± 1.4 | 0.056 |
| Deaths | 12.28% (35) | 9.70% (108) | 0.19 |

**Supplementary table 1 Comparison of HV positive and HV negative patients data in 2015 cohort**

**Table 2**

| Parameter | HV- Infected  (N=249) | Non-Infected (N=1448) | P - value |
| --- | --- | --- | --- |
| Age (years) | 60 ±12.5 | 61 ± 12.9 | 0.224 |
| Gender (male) | 57.2% (143) | 60.1% (871) | 0.373 |
| Duration of hemodialysis (years) | 8.53 ± 6.4 | 5.06 ± 4 | <0.0001 |
| Type 2 diabetes mellitus (%) | 17.6% (44) | 26.7% (388) | 0.002 |
| Coronary artery disease (%) | 71.6% (179) | 48.8% (708) | <0.0001 |
| Left ventricular hypertrophy (%) | 72.4 % (181) | 66.1% (958) | 0.050 |
| Peripheric artery disease (%) | 20.8% (52) | 22.3% (324) | 0.598 |
| Heart valve calcification | 70.4% (176) | 63.8% (924) | 0.044 |
| Parathyroidectomy | 15.2% (38) | 8% (116) | 0.0003 |
| Presence of stroke history (%) | 9.2% (23) | 9.4 % (237) | 0.920 |
| Hemoglobin (g/dl) | 11.1 ± 1.44 | 11 ± 1.4 | 0.298 |
| Ferritin (ng/ml) | 839.7 ± 641.5 | 930.8 ± 621.4 | 0.033 |
| TAST (%) | 33.8 ± 17.2 | 33.6 ± 51.5 | 0.951 |
| CRP | 1.16 ± 1.8 | 1.6 ± 3.1 | 0.029 |
| Phosphorus (mg/dl) | 4.94 ± 1.3 | 5.1 ± 1.4 | 0.092 |
| Calcium (mg/dl) | 8.78 ± 0.7 | 8.78 ± 0.69 | 0.99 |
| iPTH (pg/ml) | 621 ± 670.1 | 533.4 ± 557.9 | 0.025 |
| Albumin (g/dl) | 4.02 ± 0.4 | 4.1 ± 0.4 | 0.0003 |
| Deaths | 6.8% (17) | 12.9% (187) | 0.006 |

**Suplementary table 2 Comparison of HV positive and HV negative patients data in 2019 cohort**
